# Supplementary material for: Mapping Satellite Glial Cell Heterogeneity Reveals Distinct Spatial Organization and Implies Functional Diversity in the Dorsal Root Ganglion
Source: Adv Sci (Weinh). 2025 Oct 24;13(1):e11569. doi: 10.1002/advs.202511569 (PMC12767089; doi:10.1002/advs.202511569)
Supplement: Supplementary file 1 — Supporting Information [file ADVS-13-e11569-s001.docx]

**Table 1: Selected enriched markers from each of the clusters of the present study are also**

**enriched in specific clusters of other SGC heterogeneity datasets.**

| Marker | Ahlgreen  et al.  (present study) | Avraham  et al. ^[13]^ | Mapps  et al. ^[15]^ | Tonello  et al. ^[16]^ | Weperen  et al. ^[12]^ |
| --- | --- | --- | --- | --- | --- |
| *Fabp7* | C1 | C3 | Sens, Symp | SGCs I | C2, C3 (Mature) |
| *Glul* | C1 | C3 | Sens | SGCs I | C1 (Immature) |
| *Gja1* | C1 | C3 | Sens | SGCs I | C1 (immature) |
| *Kcnj10* | C1 | C3 | - | SGCs I | - |
| *Pou3f1* | C2 | C1 | Sens, IEG | - | C4 (Aged) |
| *Scn7a* | C3, C4 | C4 | GR, IEG | SGCs II | C4, C5 (Aged) |
| *Ifit3* | C4 | C4 | Immune | - | C6 (Immune) |

**Supplementary Figure S1: Genes expression and features of the identified subclusters.**

**
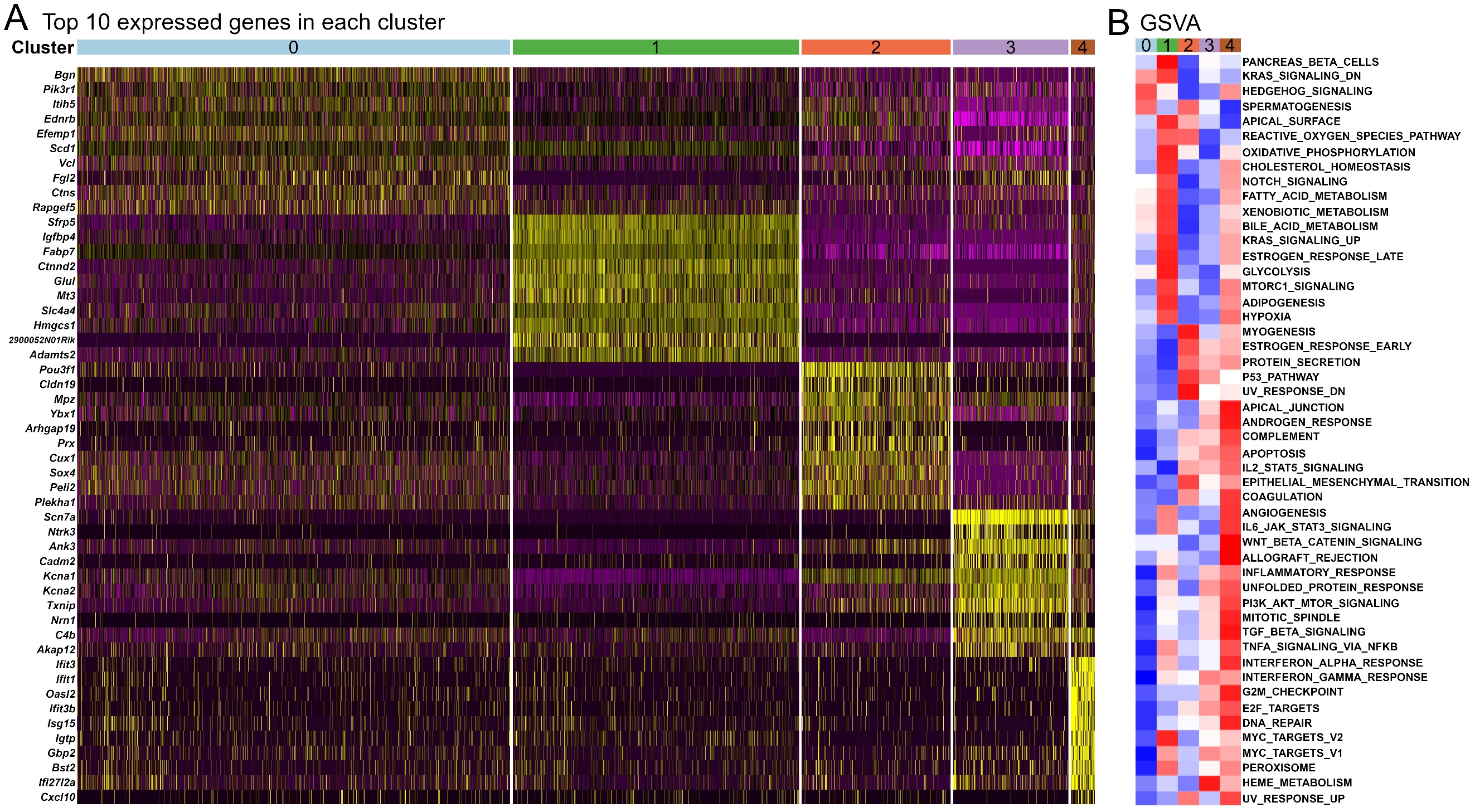
**

**Supplementary figure S2: Comparison of the top 50 highest expressed genes among the**

**clusters of five SGC heterogeneity studies.**

**
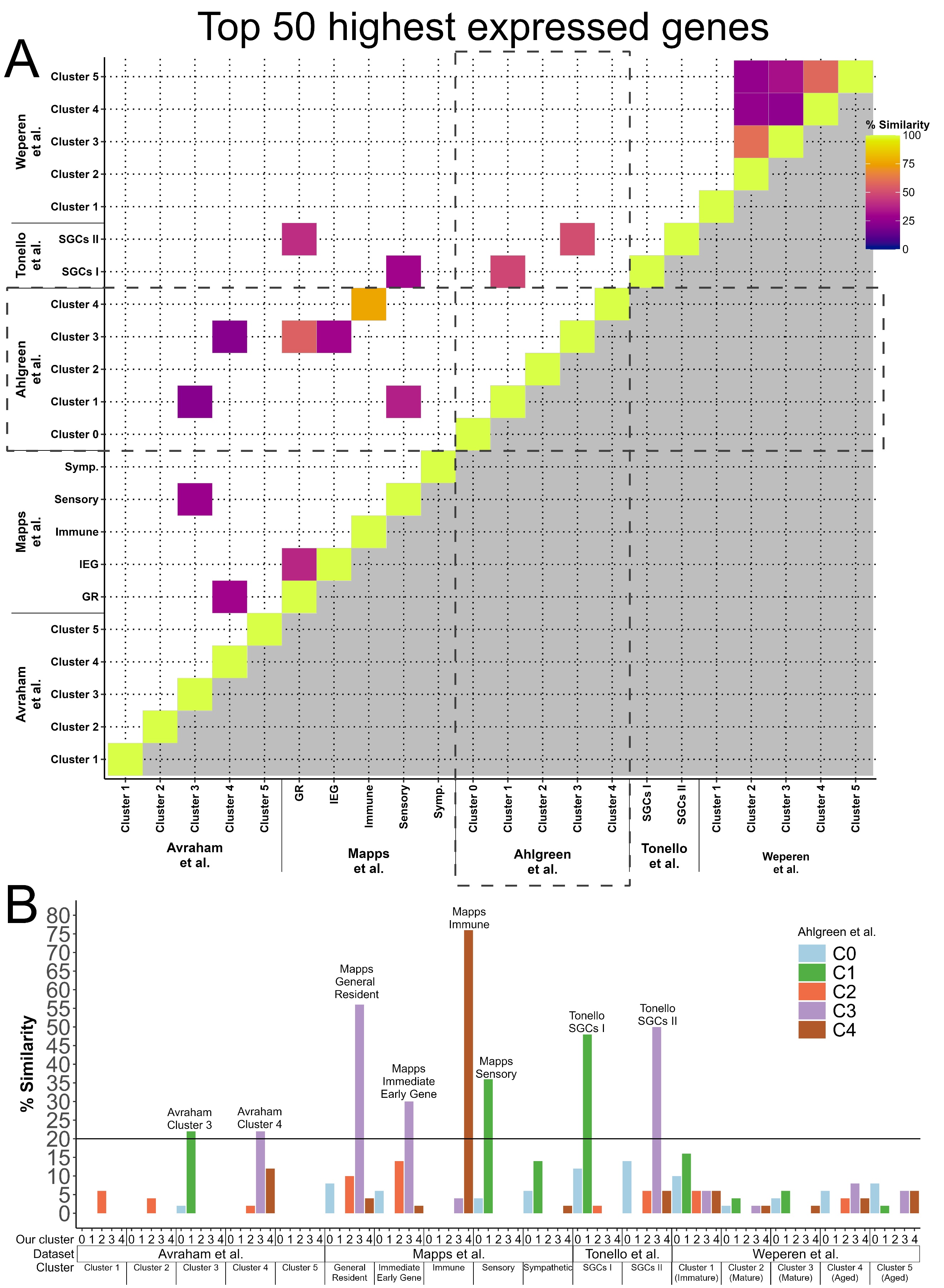
**

**Supplementary figure S3: Comparison of enriched genes among the clusters of five SGC**

**heterogeneity studies.
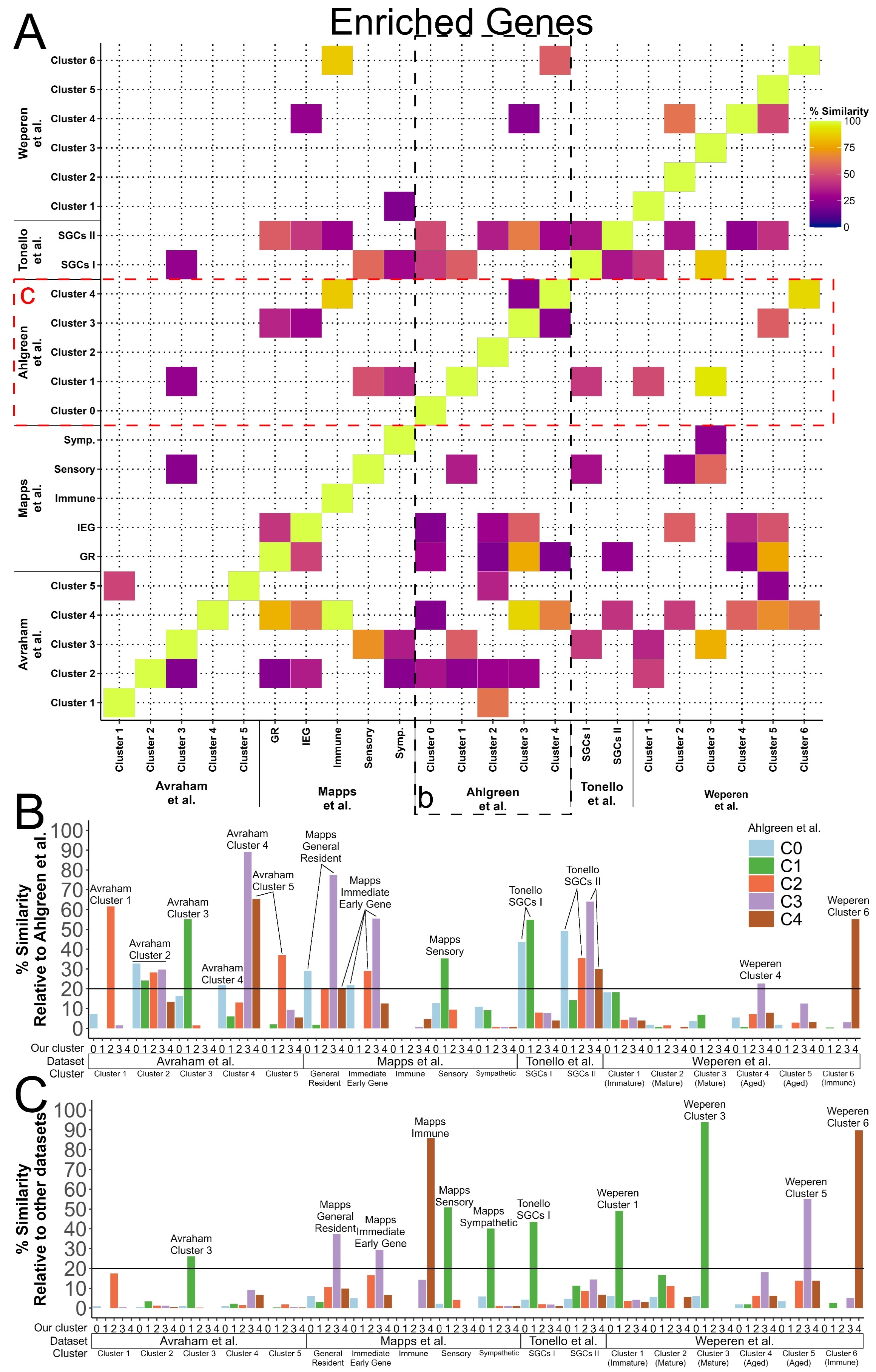
**

**Supplementary Figure S4: Further characterization of SCN7A**^+^ **SGCs**

**
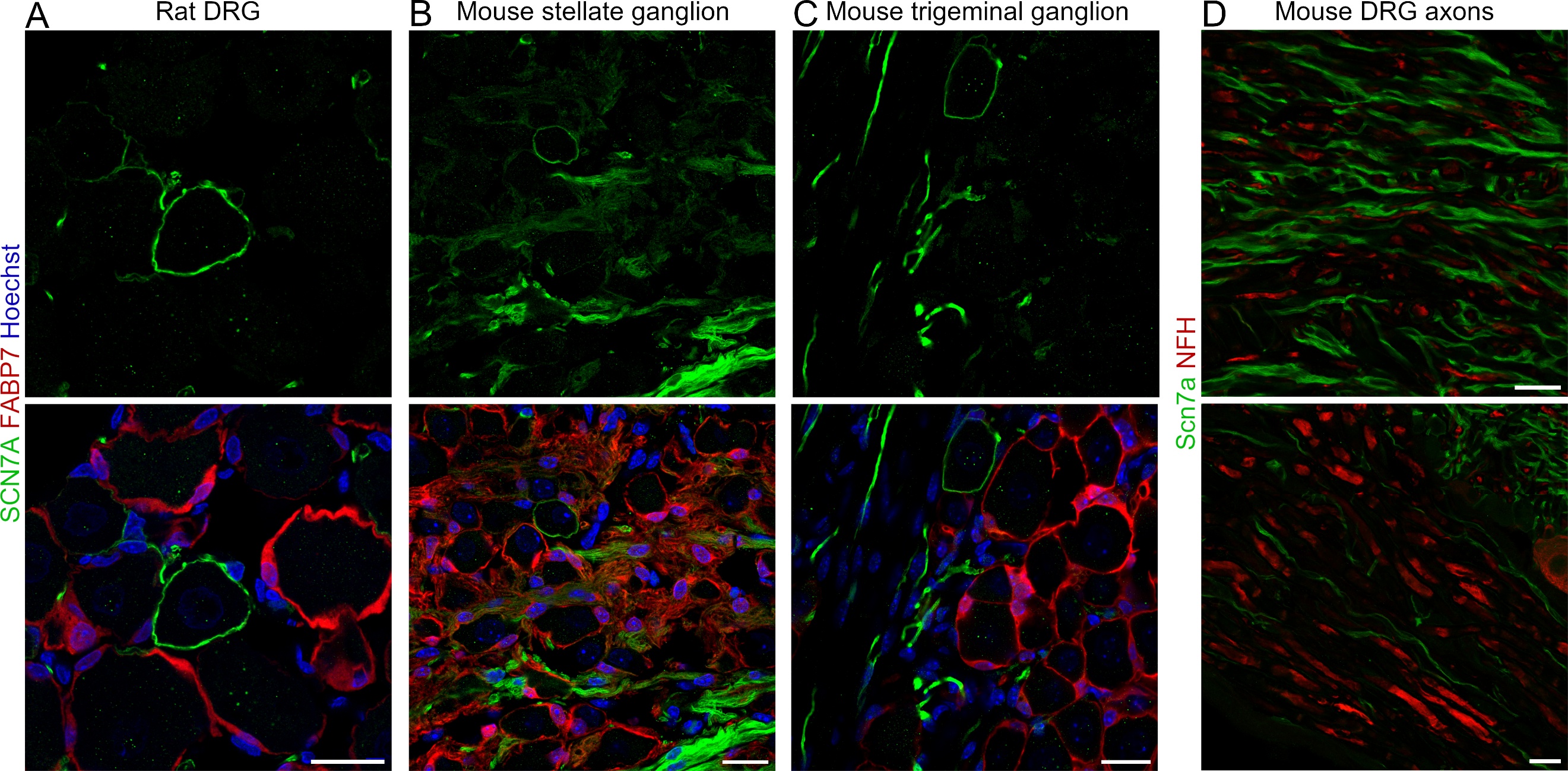
**

**Supplementary Figure S5: Comparative analysis of *Scn7a*-enriched clusters among**

**datasets from published studies and the present study.**

**
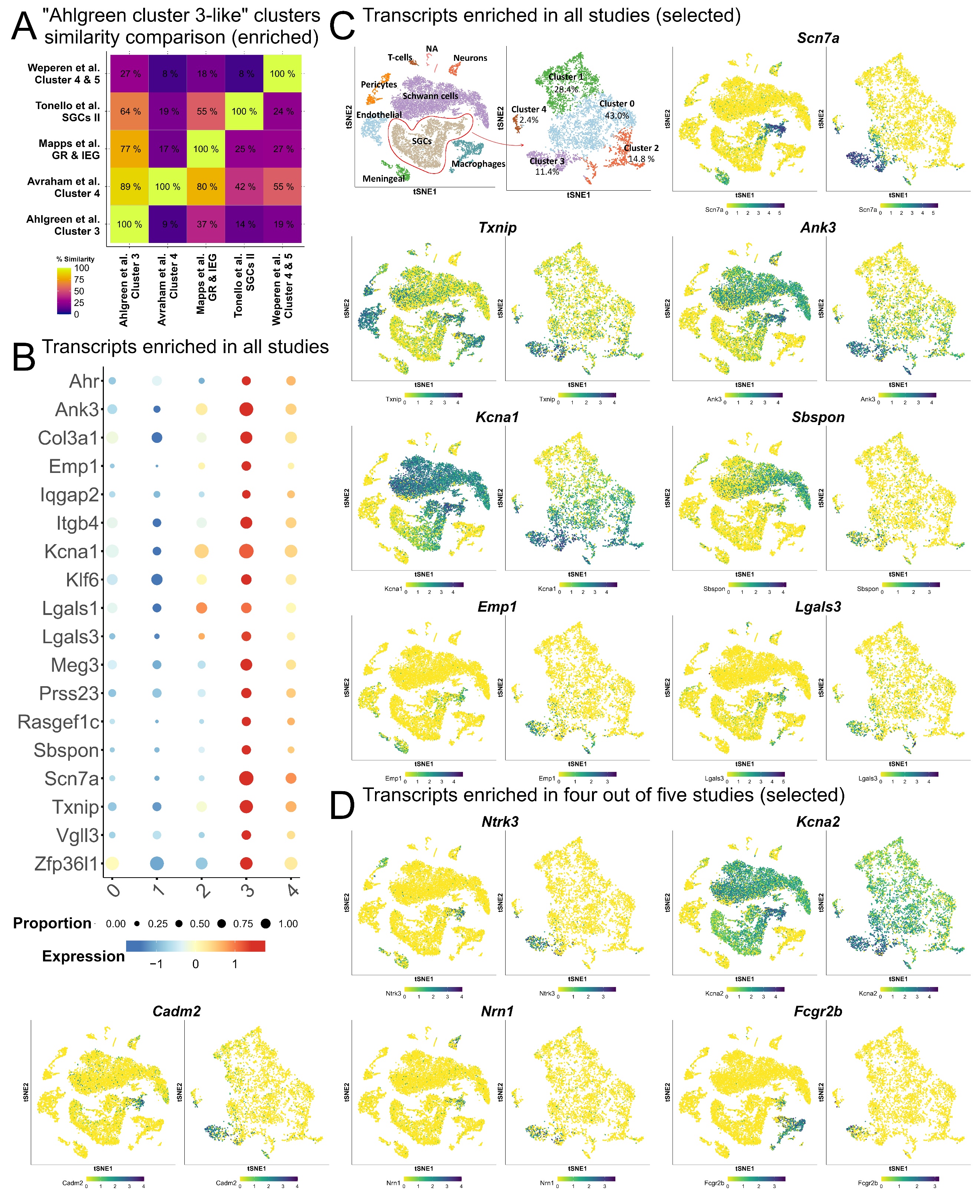
**

**Supplementary Figure S6: Automated annotation of neuronal somata in DRG sections**

**using Deepflash2.**

**
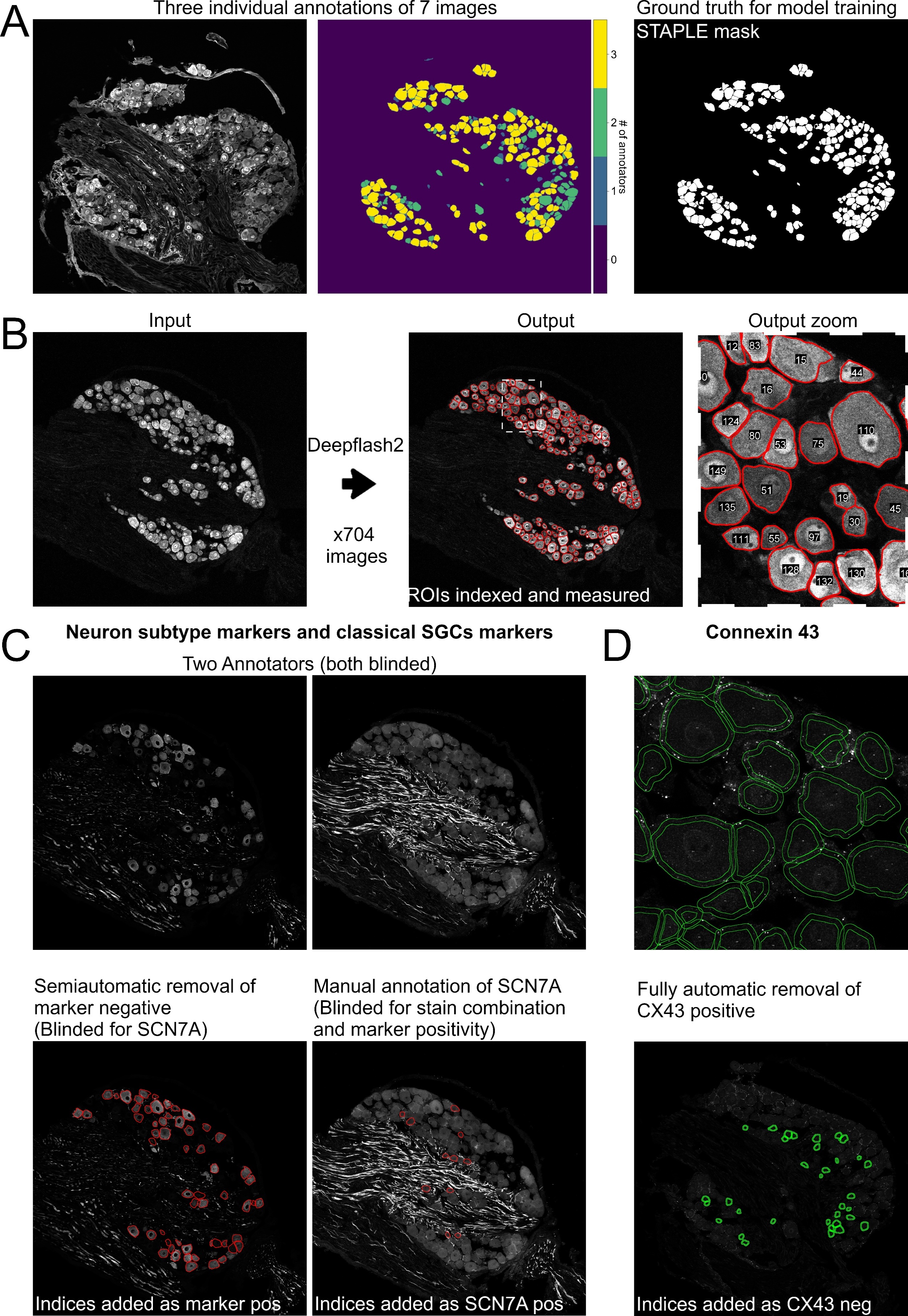
**

**Supplementary Table S1: Deepflash2 sample information.**

| Sample | # Mice | #Unique DRG/marker | #Unique DRG/mouse | # Unique neurons/marker |
| --- | --- | --- | --- | --- |
| Classical SGC markers | **14** | **50.5±7.2** | **6.8±2.2** | **11570±1393** |
| FABP7 | 6 | 49 | 7-10 | 11676 |
| KIR4.1 | 6 | 55 | 7-11 | 11731 |
| CX43 | 6 | 57 | 8-11 | 13133 |
| GS | 8 | 41 | 4-7 | 9742 |
|  |  |  |  |  |
| Neuron subtype markers | **14** | **49.3±4.6** | **6.4±2** | **10853±1865** |
| NFH | 6 | 50 | 7-9 | 10707 |
| CGRP | 6 | 46 | 5-9 | 9878 |
| SP | 6 | 52 | 8-9 | 11520 |
| TH | 6 | 51 | 7-9 | 12785 |
| IB4 | 6 | 52 | 8-9 | 11341 |
| Plexin C1 | 8 | 39 | 4-6 | 7300 |
| GFRα2 + IB4 | 8 | 43 | 4-7 | 9466 |
| OSMR + IB4 | 8 | 41 | 2-7 | 9640 |
|  |  |  |  |  |
| Combined sample   unique neurons | **#Mice** | **#Non-overlapping DRG sections** | **#Non-overlapping DRG  sections/mouse** | **#Unique neurons** |
| All neurons | **14** | **214** | **4-42*** | **48668** |
|  | | | | |

**Supplementary table S1: Deepflash2 sample information.** The combined dataset used for SCN7A+ SGC frequency quantification included a variable number of DRG sections representing each mouse, ranging between 4 and 42. No significant difference in %SCN7A+ between under- and over-represented mice (unpaired 2-tailed students t-test with equal variance: p=0.83)

**Supplementary Table S2: Human DRG donor information and medical history**

| Donor # | Age | Sex | Ethnicity | Cause of Death | DRG used | Medical History |
| --- | --- | --- | --- | --- | --- | --- |
| 1 | 29 | M | Black | Head Trauma/Blunt Injury | LX | Marijuana use, no other significant history |
| 2 | 29 | F | White | Anoxia/Drug Overdose | L2/L4 | Seizures; Bipolar; Depression; Drug Abuse (chronic opioid and Benoz); Tox positive on admission for Barbituates, Benzo, THC, and Opiates; Tubal ligation |
| 3 | 27 | M | Black | Anoxia/Cardiac Arrest | L4 | Seizures last one 2 weeks prior to admission (on meds name unknown), HTN (amlodipine x1 yr), 1 pack yr smoking hx, Tox + for THC. |
| 4 | 31 | F | White | CVA/Stroke | L3 | Neurosyphilis, HTN, Breast Cancer, Thyroid Cancer (resection), Bulging of cervical and lumbar discs, Endometriosis, Mental Disorder, Anxiety, Bipolar, Depression, STD, trichomonas, HSV; Surgical history: Cholecystectomy, Lymph Node Biopsy, Throidectomy (Partial, 2015), Uterine ablation, Skin graft back rt thigh (1997); Smoking history ~6 pack yr; Drug use Ecstacy, Tox + for Amphetamines, use since 2015; |
| 5 | 25 | F | Black | Head Trauma/Blunt Injury/MVA | L4 | On Birth Control; Previous C-Section 4/1/2018; |
| 6 | 31 | M | White | Anoxia/Drug Overdose | L5 | Asthma dx at 2 y old use of Flonase and breathing treatments; Drug screen + for Amphetamines, Benzo and THC, reported Fentanyl overdose; Rx for Xanax, reported to be addicted/abused; Recent incarceration for poss of Drugs; Reported drug use of Marijuana (15 yrs), Barbituates (12 yrs), Heroin used needles (7-8 yrs), Cocaine (ukn time), Esctacy (ukn time), Benzo (1 yr); Cigarette smoking 5 cig/day since age 15; Family hx of diabetes grandparent level; |

**Supplementary Table S3: Donor demographics table.** Abbreviations: CVA: cerebrovascular accident, MVA: motor vehicle accident.

**Supplementary Table S3: Antibodies and staining reagents.**

**Primary antibodies and staining reagents**

| **Target** | **Host** | **Clonality** | **Company** | **Product number** | **Concentration** | **RRID** |
| --- | --- | --- | --- | --- | --- | --- |
| **Classical SGC markers** | | | | | | |
| FABP7 | Gt | pAb | R&D systems | AF3166 | 1:30 (IHC)  1:25 (ISH) | AB_2100475 |
| GS | Rb | pAb | Abcam | ab49873 | 1:200 | AB_880241 |
| KIR4.1 | Gp | pAb | SySy | 472 005 | 1:500 | AB_2924995 |
| CX43 | Gt | pAb | Antibodies.com | A121557 | 1:1000 | NA |
| **Novel SGC subtype markers** | | | | | | |
| SCN7A | Rb | pAb | Novus Biological | NB100-81029 | 1:200 (IHC)  1:100 (ICW) | AB_1110316 |
| OCT6 | Rb | pAb | Genetex | GTX134063 | 1:200 | AB_2887197 |
| *Mm-Ifit3-C3*  *Fl v2 HRP-C3*  *TSA Vivid™-520* | - | - | ACD | 508251  323106  323271 | 1:50  1:1500 | NA |
| **Neuron markers** | | | | | | |
| β-III-tub | Ms | mAb (5G8) | Promega | G7121 | 1:2000 | AB_430874 |
| NEUN | Ms | mAb (A60) | Millipore | MAB377 | 1:200 | AB_2298772 |
| NEUN | Gt | pAb | Novus | NBP3-05554 | 1:500 | AB_3534041 |
| NFH | Ms | mAb (N52) | Sigma | N0142 | 1:1000 | AB_477257 |
| CGRP (N-20) | Gt | pAb | Santa Cruz | sc-8856 | 1:125 | AB_637693 |
| SP | Rt | mAb (NC1/34) | Novus | NB100-65219 | 1:20 | AB_965531 |
| TH | Sh | pAb | Novus | NB300-110 | 1:125 | AB_10002491 |
| IB4-AF647 | - | - | Invitrogen | I32450 | 2 µg/ml | SCR_014365 |
| Plexin C1 | Sh | pAb | R&D | AF5375 | 1:400 | AB_2284038 |
| GFRα2 | Gt | pAb | R&D | AF429 | 1:20 | AB_2294621 |
| OSMR | Gt | pAb | R&D | AF662 | 1:100 | AB_355511 |
| **Human DRG stainings** | | | | | | |
| SCN7A | Rb | pAb | Novus Biological | NB100-81029 | 1:200 | AB_1110316 |
| FABP7 | Gt | pAb | R&D | AF3166 | 1:30 | AB_2100475 |
| GS | Ms | mAb (GT1055) | Genetex | GTX630654 | 1:500 | AB_2888230 |
| GS | Rb | pAb | Thermo | 11037-2-AP | 1:200 | AB_2110650 |
| KIR4.1 | Rb | pAb | Alomone | APC-035 | 1:500 | AB_2040120 |
| CX43 | Rb | pAb | Cell signaling | #3512 | 1:200 | AB_2294590 |
| β-III-tub | Ms | mAb (2G10) | Sigma | T8578 | 1:1000 | AB_1841228 |
| Peripherin | Ch | pAb | Encorbio | CPCA-Peri | 1:500 | AB_2284443 |
| **Other** | | | | | | |
| p75NTR (NGFR) | Gt | pAb | R&D | AF1157 | 1:200 | AB_2298561 |
| SCN7A | Rb | pAb | Novus Biological | NBP1-87075 | 1:100 | AB_11033949 |
| Hoechst | - | - | Invitrogen | H3570 | 1:30000 | NA |
| DAPI |  |  | Cayman Chemical | #14285 | 1:5000 | NA |
| IFIT3 | Rb | pAb | Invitrogen | Pa5-22230 | - | AB_11153289 |
| ISG15 | Rb | pAb | Cell Signaling | #2743 | - | AB_2126201 |
| ISG15/UCRP | Gt | pAb | R&D | AF4845 | - | AB_2249121 |
| BST2 | Ms | mAb | ThermoFisher | #16-3172-81 | - | AB_763429 |
| BST2 | Rt | mAb | Leinco | C791 | - | NA |
| GBP2 | Rb | Rb | Proteintech | 11854-1-AP | - | AB_2109336 |
|  | | | | | | |
| **Secondary Antibodies** used for mouse tissue (Host species: Donkey, all diluted 1:300) | | | | | | |

| **Fluorophore** | **Target species** | | | | | | |
| --- | --- | --- | --- | --- | --- | --- | --- |
|  | Mouse | Goat | Rabbit | Rat | Sheep | Guinea pig | Chicken |
| Alexa Fluor 405 | Invitrogen  A48257 |  |  |  |  |  |  |
| Alexa Fluor 488 | Invitrogen  A21202 | Invitrogen  A11055 | Invitrogen  A21206 |  | Invitrogen  A11015 | Jackson immuno  706-545-148 | Invitrogen  A78948 |
| Alexa Fluor 568 | Invitrogen  A10037 | Invitrogen  A11057 | Invitrogen  A10042 | Invitrogen  A78946 |  |  |  |
| Alexa Fluor 647 | Invitrogen  A31571 | Invitrogen  A21447 | Invitrogen  A31573 |  |  |  | Invitrogen  A78952 |

| **Secondary antibodies** used for human DRG stainings | | | | | |
| --- | --- | --- | --- | --- | --- |
| Antibody |  |  |  |  |  |
| Goat α-Mouse IgG2a, Alexa Fluor 488 |  | Invitrogen | A-21131 | 1:300 |  |
| Goat α-Mouse IgG1, Alexa Fluor 647 |  | Invitrogen | A-21240 | 1:300 |  |
| Goat α-Rabbit , Alexa Fluor 568 |  | Invitrogen | A-11011 | 1:300 |  |

**Supplementary table S3: Antibodies and staining reagents.** Abbreviations: * Gt: Goat – Ms: Mouse – Rb: Rabbit – Rt: Rat – Gp: Guinea pig – Sh: Sheep – Ch: Chicken – Dk: Donkey – pAb: polyclonal antibody – mAb: monoclonal antibody – IHC: immunohistochemistry – ICW: integrated co-detection workflow (in situ hybridization with IHC)

**Supplementary Table S4: Single cell RNA sequencing sample information**

| **DRG cell clustering** | | | | | | |
| --- | --- | --- | --- | --- | --- | --- |
| **nCells** | **Sample 1** | **Sample 2** | **Sample 3** | **Sample 4** | **Sample 5** | **Total** |
| Endothelial | 300 | 266 | 213 | 289 | 323 | 1391 |
| Macrophages | 114 | 321 | 282 | 410 | 373 | 1500 |
| Meningeal | 92 | 110 | 86 | 216 | 210 | 714 |
| NA | 32 | 86 | 51 | 43 | 65 | 277 |
| Neurons | 45 | 22 | 208 | 61 | 44 | 380 |
| Pericytes | 143 | 166 | 117 | 175 | 205 | 806 |
| Schwann_cells | 1471 | 1578 | 1195 | 1520 | 1733 | 7497 |
| SGC | 1133 | 1055 | 733 | 1338 | 1192 | 5451 |
| T-cells | 11 | 24 | 31 | 59 | 44 | 169 |
| Total | 3341 | 3628 | 2916 | 4111 | 4189 | 18185 |
|  |  |  |  |  |  |  |
| **% Cells** | **Sample 1** | **Sample 2** | **Sample 3** | **Sample 4** | **Sample 5** | **Total** |
| Endothelial | 9.0% | 7.3% | 7.3% | 7.0% | 7.7% | 7.6% |
| Macrophages | 3.4% | 8.8% | 9.7% | 10.0% | 8.9% | 8.2% |
| Meningeal | 2.8% | 3.0% | 2.9% | 5.3% | 5.0% | 3.9% |
| NA | 1.0% | 2.4% | 1.7% | 1.0% | 1.6% | 1.5% |
| Neurons | 1.3% | 0.6% | 7.1% | 1.5% | 1.1% | 2.1% |
| Pericytes | 4.3% | 4.6% | 4.0% | 4.3% | 4.9% | 4.4% |
| Schwann_cells | 44.0% | 43.5% | 41.0% | 37.0% | 41.4% | 41.2% |
| SGC | 33.9% | 29.1% | 25.1% | 32.5% | 28.5% | 30.0% |
| T-cells | 0.3% | 0.7% | 1.1% | 1.4% | 1.1% | 0.9% |
| Total | 100.0% | 100.0% | 100.0% | 100.0% | 100.0% | 100.0% |
|  |  |  |  |  |  |  |
| **SGC annotated cells** | | | | | | |
| **nCells** | **Sample 1** | **Sample 2** | **Sample 3** | **Sample 4** | **Sample 5** | **Total** |
| Cluster 0 | 475 | 452 | 314 | 555 | 547 | 2343 |
| Cluster 1 | 331 | 294 | 215 | 392 | 317 | 1549 |
| Cluster 2 | 166 | 151 | 103 | 218 | 169 | 807 |
| Cluster 3 | 123 | 136 | 82 | 138 | 143 | 622 |
| Cluster 4 | 38 | 22 | 19 | 35 | 16 | 130 |
| Total | 1133 | 1055 | 733 | 1338 | 1192 | 5451 |
|  |  |  |  |  |  |  |
| **% Cells** | **Sample 1** | **Sample 2** | **Sample 3** | **Sample 4** | **Sample 5** | **Total** |
| Cluster 0 | 41.9% | 42.8% | 42.8% | 41.5% | 45.9% | 43.0% |
| Cluster 1 | 29.2% | 27.9% | 29.3% | 29.3% | 26.6% | 28.4% |
| Cluster 2 | 14.7% | 14.3% | 14.1% | 16.3% | 14.2% | 14.8% |
| Cluster 3 | 10.9% | 12.9% | 11.2% | 10.3% | 12.0% | 11.4% |
| Cluster 4 | 3.4% | 2.1% | 2.6% | 2.6% | 1.3% | 2.4% |
| Total | 100.0% | 100.0% | 100.0% | 100.0% | 100.0% | 100.0% |

**Supplementary Table S4.** Single cell RNA sequencing sample information.

Cell numbers and frequencies of annotated DRG cell clusters and SGC subclusters of the single cell RNA sequencing dataset.
